# De Novo Synthesized Estradiol: A Role in Modulating the Cerebellar Function

**DOI:** 10.3390/ijms21093316

**Published:** 2020-05-07

**Authors:** Cristina V. Dieni, Samuele Contemori, Andrea Biscarini, Roberto Panichi

**Affiliations:** 1 Department of Ophthalmology, University of Alabama at Birmingham, Birmingham, AL 35294, USA; 2 Centre for Sensorimotor Performance, School of Human Movement and Nutrition Sciences, The University of Queensland, Brisbane 4072, Australia; s.contemori@uqconnect.edu.au; 3 Department of Experimental Medicine, Section of Physiology and Biochemistry, University of Perugia, 06129 Perugia, Italy; andrea.biscarini@unipg.it

**Keywords:** neurosteroids, plasticity, cerebellum, Purkinje cell, vestibulo-ocular reflex, estradiol, aromatase, motor control, cerebellar-dependent behavior, synaptic transmission

## Abstract

The estrogen estradiol is a potent neuroactive steroid that may regulate brain structure and function. Although the effects of estradiol have been historically associated with gonadal secretion, the discovery that this steroid may be synthesized within the brain has expanded this traditional concept. Indeed, it is accepted that de novo synthesized estradiol in the nervous system (nE2) may modulate several aspects of neuronal physiology, including synaptic transmission and plasticity, thereby influencing a variety of behaviors. These modulations may be on a time scale of minutes via non-classical and often membrane-initiated mechanisms or hours and days by classical actions on gene transcription. Besides the high level, recent investigations in the cerebellum indicate that even a low aromatase expression can be related to the fast nE2 effect on brain functioning. These pieces of evidence point to the importance of an on-demand and localized nE2 synthesis to rapidly contribute to regulating the synaptic transmission. This review is geared at exploring a new scenario for the impact of estradiol on brain processes as it emerges from the nE2 action on cerebellar neurotransmission and cerebellum-dependent learning.

## 1. Introduction

**Figure 1:**
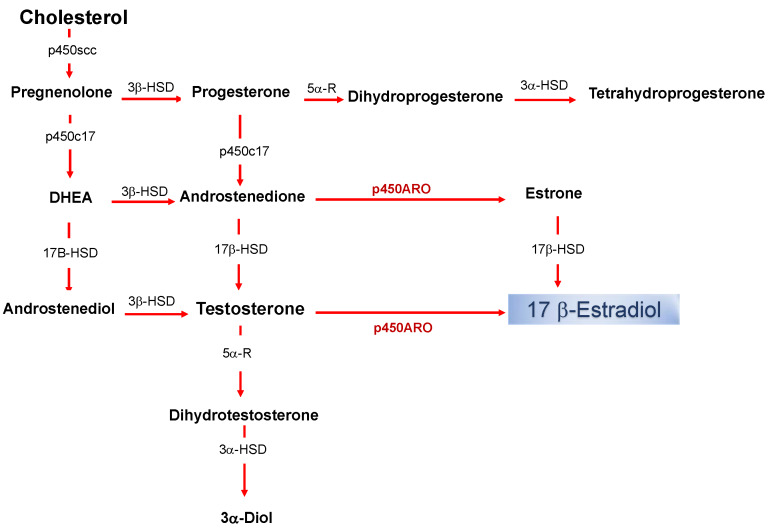
Biosynthetic pathway for neurosteroids in the brain. The arrows indicate biosynthetic pathways of neurosteroids identified in the brain. P450scc, Cytochrome P450 cholesterol side-chain cleavage enzyme; p450c17, cytochrome P450 17a-hydroxylase/C17; DHEA, dehydroepiandrosterone; 17β-HSD, 17beta-hydroxysteroid dehydrogenase; 3β-HSD 3beta-hydroxysteroid dehydrogenase D5–D4 isomerase; 5α-R, 5alfa-reductase; p450ARO, cytochrome P450 aromatase; 3α-HSD 3alfa-hydroxysteroid dehydrogenase D5–D4 isomerase.

Estrogens are part of the neuroactive steroid family that may regulate the structure and function of neural networks via multiple modes and time courses. The most potent estrogen in influencing brain functions is the 17 beta-estradiol that exerts its effect via both classical long term actions on genomic mechanisms and rapid non-classical effects [1,2,3,4,5,6,7]. The estradiol impacts on neural physiology depend on its bioavailability in a brain structure, and it has been shown that, in addition to peripheral synthesis such as in the gonads, the estradiol can be locally produced in the nervous system [8]. The de novo synthesized 17 beta-estradiol in nervous tissues, defined as neurosteroid (nE2), has the same structure and mechanisms of synthesis than the gonadal derived estrogen (Figure 1).

**Figure 2:**
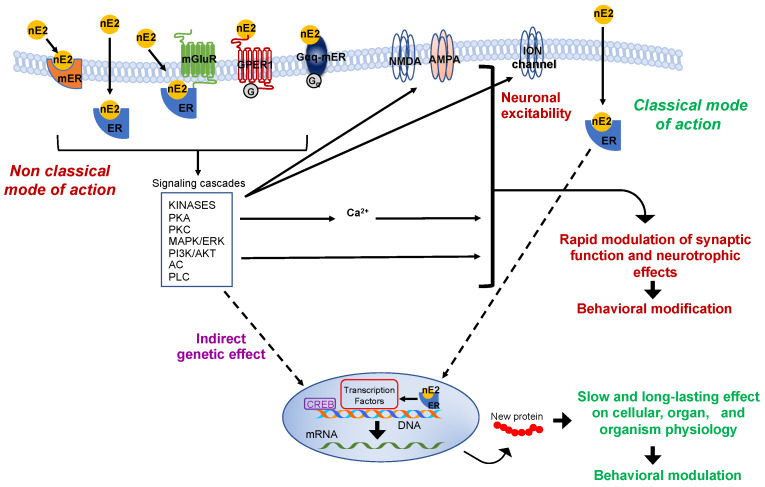
Schematic representation of the classic and non-classic mode of action of the estrogen estradiol. The schema represents possible estradiol effects on neural targets. Both classical and non-classical effects require estrogen receptors activation by estradiol (nE2). In the classic genomic mode of action, nE2 binds to cytoplasmatic estrogen receptors beta or alpha (ERs: ERα, ERβ), receptors dimerize (not shown), and translocate to the nucleus. Once bound specific estrogen response element on the DNA, the dimer possibly recruits transcriptional coregulator to modulates the gene transcription. The results may be a slow and long-lasting effect on cells and, ultimately, on the entire organism. In the non-classical mode of action, nE2 binds cytoplasmatic or membrane-associated estrogen receptors (ERs, GPER-1, Gαq). The binding triggers intracellular signaling cascades involving several kinases (e.g., PCA, Protein kinase A; PKC, Protein kinases C; MAPK, mitogen-activated protein kinase; PI3K, phosphoinositide 3-kinase), adenylyl cyclase (AC), phospholipase C (PLC), and fluctuation in intracellular calcium concentration. Such intracellular signaling may result in the rapid modulation of synaptic function (the schema also shows possible neurotrophic effects) and, ultimately, in behavioral modifications. Finally, in the estrogen-mediated indirect genetic effect, nE2 binding to estrogen receptors induces signaling cascades that might activate transcription factors (e.g., CREB, cAMP response element-binding protein) to regulate the gene transcription. AKT, Protein kinase B; AMPA, ionotropic glutamate receptor; ER, classical estrogen receptor; ERK, extracellular signal-regulated kinase; GPER-1, G protein-coupled transmembrane estrogen receptor-1; Gαq-mER, Gαq-coupled membrane-associated estrogen receptor; mER, membrane-associated classical estrogen receptor; mGluR, metabotropic glutamate receptor; NMDA, ionotropic glutamate receptor.

The production of nE2 requires an aromatase-dependent conversion of testosterone, which may be either of the peripheral origins or locally synthesized from the precursor cholesterol [9,10,11,12]. Thus, estradiol is no longer merely considered a hormone produced by the ovaries and only related to the control of female sexual maturation and reproduction. Instead, it is now known to have multiple homeostatic roles, and in the nervous system, estradiol controls a variety of processes in males as well as females [2,13,14,15,16]. Via the “classical” genomic mode of action, estradiol interacts with intracellular receptors to influence transcriptional pathways and regulate DNA transcription within minutes (Figure 2) [17].

Two typical estrogen receptor isoforms (ERs: ERα, ERβ) are known to participate in classical influence. However, to produce detectable functional effects on the cellular level or the entire organism, the “classical” mode of action requires post-transcriptional events that take hours to days to occur [7,18,19]. In the brain, faster non-classical and often membrane-initiated mechanisms are observable within a few seconds (for some electrophysiological effects) to minutes after estradiol activated signaling involving intracellular cascades that may include several protein kinases and result in cytoplasmatic fluctuations of calcium concentration (Figure 2) [19,20,21,22,23,24]. Some of these intracellular signalings could also affect the gene transcription through the so-called “indirect genetic effect” [25]. However, the essential point considered here is that estradiol may rapidly modulate pathways associated with the regulation of neurotransmission and neuronal activity such as those related to cytoplasmatic protein kinases cascades and calcium fluctuation (Figure 2) [23,26].

Rapid membrane-initiated effects involve estrogen G-protein-coupled receptors (GPCRs: GPER-1 and Gαq-mER), classical estrogen receptors (ERα, ERβ), and probably other putative receptors (e.g., ER-X) [27,28,29,30,31,32,33,34,35]. It is noteworthy to mention that non-classical estrogen actions on neural circuits are not limited to those that occur via the activation of membrane-associated estradiol pathways, but also include mechanisms that can depend on the estrogen binding of cytoplasmatic receptors with the consequent activation of various intracellular signaling cascades (Figure 2) [36].

It has been established that nE2 may be a potent regulator of the neuronal functions when produced in large amounts [4,37]. However, how generalized nE2 synthesis is within the nervous system and whether some of the nE2 effects can be even mediated by low estradiol production are issues of rising interest in neurophysiology and neuroendocrinology.

Recent evidence showed that besides the high level of synthesis, even alleged minute and localized nE2 production might contribute to rapidly modulating aspects of neuronal physiology like the synaptic transmission and plasticity (Figure 2).

**Figure 3:**
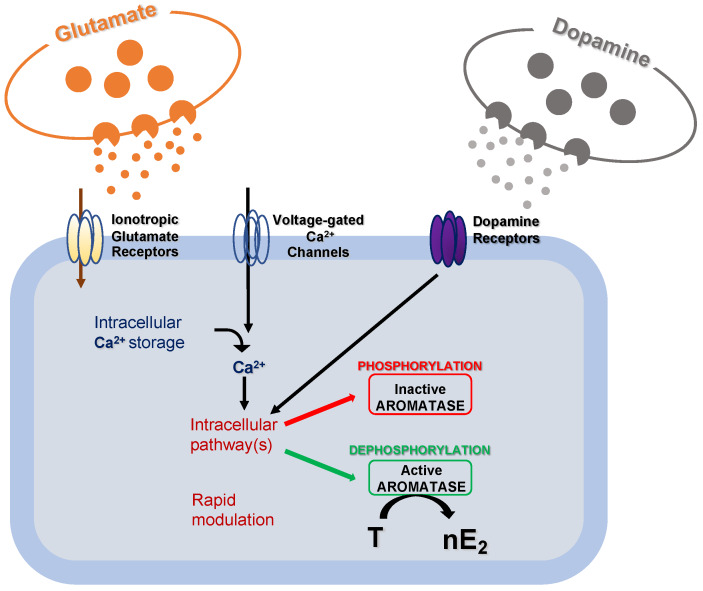
Schematic cartoon representing the post-translational mechanisms modulating aromatase activity. Fluctuations in intracellular calcium (Ca^2+^) concentration, along with glutamatergic and dopaminergic signals, might rapidly modulate intracellular pathways to regulate aromatase activity via phosphorylation/dephosphorylation processes. As a result, the transformation rate of testosterone (T) into 17 beta-estradiol (nE2) may be rapidly regulated.

Experiments conducted in the brainstem and cerebellum indicate that despite the supposed low synthesis in the experimental models used, nE2 may contribute to rapidly modulating neurotransmission via dynamic change of its availability at a synaptic level [38,39,40,41,42]. It has already been demonstrated that modifications in local estradiol synthesis may be achieved by changes in aromatase activity. Although possible changes in aromatase activity result from transcriptional control that alters the enzyme concentration, these changes generally occur slowly in a time scale of hours or days. Alternatively, much more rapid changes in the rate of nE2 production may result from post-translational events that potentiate/inhibit the enzyme activity (Figure 3) [38].

Post-translational modifications are common mechanisms involved in the control of protein activity in the brain, like in the case of the regulation of neurotransmitter activity in neurons [43].

The evidence introduced above provides new insight into the effectiveness of nE2 in regulating neuronal activity and indicating that estradiol may play an essential role in the modulation of network functioning, even in brain structures in which, so far, its synthesis has been assumed to be too low to be effective. Here, we review the emerging view from this scenario that expands the traditional significance of nE2 production into a new physiological role and includes recently suggested mechanisms involved in the rapid control of the neural transmission, with particular regard to the synaptic transmission in the cerebellum.

## 2. The Effectiveness of the De Novo Synthesized Estradiol in Rapidly Influencing the Neuronal Functioning

It has long been assumed that the impact of nE2 on the regulation of neural functions should be restricted at those brain areas in which the estradiol synthesizing enzyme aromatase is highly expressed and, of course, estrogen receptors are present. According to this assumption, in such areas, nE2 may rapidly reach high concentrations [4,44,45], bind membrane ERs, or activate other non-classical mechanisms to initiate intracellular signaling and acutely influence the neuronal physiology [13,21,33,36,46]. Rapid activation of membrane estrogen pathways by nE2 may be facilitated by the co-localization of aromatase and estrogen receptors in the plasma membrane or their localization at the synaptic level in neurons of the central nervous system [47,48,49]. Non-classical influences include, but are not limited to, the modulation of synaptic activity, synaptogenesis, and spinogenesis (Figure 2) [23,50,51]. Rapid functional regulation of synaptic activity by estradiol has been found, so far, for the glutamatergic, GABAergic, cholinergic, and dopaminergic systems [27,41,42,52,53,54,55,56,57]. Of course, also classical genomic delayed effects influencing neuronal structure and function can be mediated by nE2 and interact with the non-classical mode of actions [2,13,19]. nE2 may, therefore, regulate over different timescale phenomena such as synaptic transmission, spine density, synaptic connectivity, synaptogenesis, and neurogenesis, that mediate behavioral changes (Figure 2) [13,20,21,27,58,59].

In many vertebrates, brain areas with the highest aromatase expression include hypothalamic preoptic nuclei, the bed nucleus of the stria terminalis, and medial amygdala, typically involved in the control of stress responses, sexual and social behaviors [2,60,61,62] and other structures that, like in the case of the hippocampus and inferior olive, are related to sensorimotor aspects of information processing and learning phenomena [44,63,64,65,66]. Consistently, besides its role in the regulation of reproduction and sexual functions [1,2,67,68,69], nE2 may influence a variety of other processes and behaviors from the mood to learning and memory formation and have a neuroprotective role as well [14,27,50,52,70,71,72,73,74,75,76,77,78].

However, despite a significant interspecific variability, low aromatase expression may be present in other brain areas such as temporal, occipital, and cingulate cortex, putamen, brainstem, and cerebellum of adult animals including rodents and humans [66,79,80,81,82].

Rodents are the most used model to study brain functions. For instance, in the cerebellum of rodents, aromatase is highly expressed only during neonatal life while it is maintained at a constant low level during peripubertal (the period between day 30 and day 45 after birth) and adulthood (post-pubertal) [66,83,84]. Thus, while the role of nE2 in regulating cerebellar development has been investigated [85,86], its effects upon the functioning of the cerebellum in adults (in the following text, the term “adult” refers to a period comprehensive the peri and post-pubertal period) have long been considered irrelevant or ignored.

Nevertheless, the age-related maintenance of a low level of aromatase expression in the cerebellum seems to have a cardinal physiological significance in the contribution of controlling the cerebellar function. Recent reports showed that nE2 synthesis rapidly influences the glutamatergic transmission between the parallel fibers and Purkinje cells in the cerebellar cortex and acutely impact cerebellar dependent behaviors in adult rodents [39,40,74,87]. Furthermore, the high level of estrogen receptors found in the cerebellum of adults is supportive of cerebellar responsiveness even to a minute and localized nE2 synthesis [88,89,90]. Table 1 summarizes the main findings showing the aromatase and estrogen receptors distribution into the cerebellar cortex in adult as well as neonatal rodents.

The above-reported findings are consistent with previous discoveries indicating an acute modulation of nE2 on glutamatergic synapses in vestibular nuclei, which are assimilable to extra cerebellar nuclei and have, like the cerebellum, a low level of aromatase expression [41,42,66,91,92,93].

Thus, even at low expression, aromatase may play a functional role in allowing the brain to exert dynamic control over local neuronal functions via nE2 synthesis, as may be the case of synaptic transmission and plasticity in the cerebellar circuitry.

## 3. Significance of the nE2 Influence upon Cerebellar Functioning

A relatively simple model that offers the unique opportunity to study neuronal aspects responsible for cerebellar-dependent learning is provided by the adaptation of the vestibulo-ocular reflex (VOR), a gaze stabilizing reflex which makes possible clear vision during head movements. VOR adaptation is mediated by combining visuo-vestibular stimulation, and it is an essential process to maintain calibrate and effective the reflex [106,107,108,109]. Furthermore, adaptive increases (gain up) and decreases (gain down) in the magnitude of eye movements in VOR can be experimentally induced by coupling specific visual-vestibular stimuli [74,110,111]. Most important, the VOR model provides the advantage to examine a motor behavior that strictly depends on a well-known circuitry in which the nE2 production can be deficient but localized over the synaptic system essential for the cerebellar learning [62,112,113,114,115,116]. In other words, the VOR model is an ideal arena to study the efficacy of nE2 in modulating neurotransmission and related behavior.

**Figure 4:**
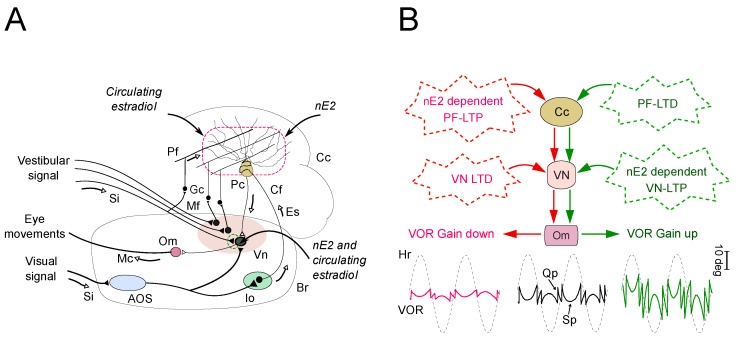
Impact of estradiol on plasticity sites in the vestibulo-cerebellar network. (**A**) Schematic representation of the neural connections mediating the VOR and possible sites of influence of circulating estradiol and nE2 (dashed lines); (**B**) Effect of nE2 on the plasticity mechanisms that could be responsible for the adaptive modification of the VOR. The red and green pathways indicate the modifications in the VOR circuitry underlying the gain down and gain up adaptation, respectively. In the bottom are reported representative VOR position traces recorded by an infrared technique in adult rats during horizontal head rotation (Hr, black dashed traces) in the dark in the control condition (black) and after adaptation paradigms (red and green). AOS, accessory optic system; Br, brainstem; Cc, cerebellar cortex; Cf, climbing fiber; Em, extra-ocular muscles; Es, error signal; Gc, granule cell; Io, inferior olive; Mc, Motor command; Mf, mossy fibers; nE2, neurosteroid estradiol; Om, ocular motoneurons in the midbrain; Pc Purkinje cell; Pf, parallel fiber; PF-LTP, LTP at the parallel fiber-Purkinje cell synapse; PF-LTD, LTD at the parallel fiber-Purkinje cell synapse; Qp, quick eye movement phases; Si, sensory inflow; Sp, slow eye movement phases; Vn, vestibular nuclei; VN-LTP, LTP in the vestibular nuclei; VN-LTD, LTD in the vestibular nuclei; VOR, vestibulo-ocular reflex.

The VOR is mediated by a direct neural pathway through the vestibular nuclei in the brainstem and an additional more complex loop within the flocculus and paraflocculus of the cerebellum. The loop includes glutamatergic axons from granular cells, known as parallel fibers, that synapse onto Purkinje cells (PCs) in the cerebellar cortex, and inhibitory GABAergic projections from PCs to the direct vestibular pathway [113,117,118,119,120] (Figure 4).

Therefore, increases in the efficacy of inhibitory projections from PCs would result in a corresponding decrease in the amplitude of the downstream signal transmitted through the direct pathway controlling the VOR. In contrast, reductions in inhibitory projections would result in a corresponding increase in the downstream signal (Figure 4). Consistently, experimental evidence indicates that the leading candidate mechanisms associated with memory encoding in VOR adaptation are the long-term plasticity (potentiation and depression) at the parallel fiber-PC synapses (Figure 4) [112,121,122,123]. Other synaptic plasticity sites that may play a late role in VOR memory consolidation could be present in the direct pathway in vestibular nuclei (Figure 4) [117,118,124,125]. Notably, as mentioned above, it has recently been shown that in adult rodents, nE2, as well as a high plasma concentration of estrogen, may similarly modulate the neuronal transmission at specific synapses into the VOR network as it is the case of the synapse between parallel fibers and PCs [39,40,41,42,87,91,97,126]. Moreover, the evidence that nE2 synthesis may also rapidly regulate the encoding of VOR adaptation and some other motor behavior controlled by the cerebellum is consistent with a nE2 modulation upon the cerebellar network [39,40,74,122].

The finding that cerebellar neural pathways are responsive to nE2 in adult rodents has relevance for the potential impact that the de novo estradiol syntheses may exert on the control of multiple aspects of cerebellar functioning after the neonatal period. Indeed, considering the homogeneity of structures and synaptic organization throughout different cerebellar regions that contribute to modulate various sensory-motor aspects related to movement and even non-motor phenomena [120,127,128,129], common nE2 modulatory mechanisms could be shared across the cerebellum to influence a variety of processes and functions. For instance, the effects of estradiol observed on the regulation of cerebellar neurotransmission and related to the modulation of learning in the VOR [40,97] were found in vermis, a cerebellar structure external to the circuitry mediating the vestibulo-ocular reflex [120,122].

Understandings the mechanisms underlying the nE2 action on cerebellum in rodent models could have an impact on human health, offering potential insights into the possibility of developing innovative intervention strategies in diseases-involving cerebellar abnormalities [130,131,132,133,134] and supporting neuroprotection [135,136]. The similarity of the estradiol pathway in the cerebellum and brainstem between rodents and humans [66,79,80], indeed, suggests similar action mechanisms of nE2 upon cerebellar networks in these species. Furthermore, it is reasonable to assume that a role of nE2 in controlling neuronal functioning via modulation of synaptic transmission, similar to that detected in the cerebellum, could also be extended to other brain regions where so far, due to low aromatase expression, estradiol synthesis has been not identified or considered not functionally relevant.

## 4. The Impact of nE2 upon Cerebellar Functioning in Adult Rodents: Multiple Lines of Evidence

The discovery that cerebellar PCs are one of the main sites of neurosteroidogenesis in the brain of neonate mammals has provided the opportunity to investigate the impact of nE2 in regulating brain neural networks develop during neonatal life [85,137]. One of the best-known effects of nE2 in the developing cerebellum includes the modulation of dendritic growth, spinogenesis and, synaptogenesis via activation of estrogen cognate nuclear receptor (mostly ERβ) in PCs. Such nE2 effect is a “classical” delayed genomic action mediated by the neurotrophic factor BDNF and involving Purkinje and external granular cells, both expressing a high level of aromatase [84,85,86,114,137].

Notably, in the cerebellum of adult rodents, the expression and distribution of the enzymatic machinery to produce estradiol, and the distribution of estrogen receptors drastically change compared to a neonatal animal (Table 1). As discussed above, aromatase expression becomes very low, with the enzyme substantially restricted to PCs [66,83,84]. ERαs are expressed at a negligible level and primarily localized within the PCs nucleus and cytoplasm [88,89,100]. The expression of ERβs is kept high with the receptors present in the soma (cytoplasm and nucleus) and dendrites of Purkinje cells and mostly in the cytoplasm of granular, Golgi, and basket type neurons [88,89,90,97,101,105]. GPER-1 is also present in the cerebellum with high, moderate, and low expression in PCs, granular cell layer, and molecular layer, respectively. However, its potential role in cerebellar functioning is almost unexplored [29]. To our knowledge, no information is available on the presence in the adults’ rodent’s cerebellum of estradiol membrane and the G-protein-coupled receptors ER-X and Gαq-mER, respectively. However, may the reorganization of the estradiol pathway into the cerebellar cortex during adulthood still indicate a functional relevance for nE2 in controlling neuronal functions? Very likely, the answer to this question is yes. This type of nE2 pathway reorganization in adults suggests that estradiol may be locally synthesized and potentially exert influence, at least, at the level of synaptic transmission as it could be the case for the synapses converging onto PCs. Furthermore, based on ERs distribution, this influence should possibly be mediated via PCs, granular, and Golgi cell ERβs or PCs-granular cells GPER-1 activation. Although the studies of the impact of nE2 in regulating cerebellar neuronal activity are only at the beginning, some general rules describing the nE2 action on the cerebellum are emerging.

The works of Smith and colleagues first demonstrated the involvement of estrogen in influencing the glutamatergic neurotransmission between parallel fibers and PCs by applying estradiol directly to the cerebellum or administering it via subcutaneous injection in the adult ovariectomized rat [138]. Further, these studies showed that locomotor-induced Purkinje cell activity could be affected by either administration of estradiol or high level of circulating estrogen during proestrus [126,139]. Afterward, Andreescu et al., showed that endogenously induced high level of plasma estradiol in ovariectomized mice might affect long-term potentiation at the parallel fiber to Purkinje cell synapse without impacting the long-tern depression and basal neurotransmission. Furthermore, they found that the high level of plasma estradiol increased the size of the postsynaptic complex at the parallel fiber to PC synapse and regulated VOR adaptation through activation of ERβs signaling in PCs [97]. Interestingly, the authors did not observe the modulatory effects of estradiol on the synapses between climbing fiber and PCs. Besides, they showed differences in VOR adaptation during the natural estrous cycle. Thus, the authors pointed to the importance of the gonadal-dependent plasma estradiol fluctuation in modulating cerebellar functioning [97].

However, the initial concept that the cerebellum might be a mere target of circulating hormones has been recently further developed to extend the action of estrogen into the adult cerebellum to the de novo synthesized estradiol . Hedges et al. demonstrated that nE2, as well as gonad-derived estradiol (at high plasma level), might regulate parallel fiber-Purkinje cell synapses transmission by enhancing the glutamatergic signaling in the adult of both male and female mouse [39,87]. Using in vivo imaging and in vitro electrophysiological techniques, they found that the effect of estradiol on glutamatergic neurotransmission was mainly mediated by an estrogen-dependent activation of metabotropic glutamate receptors type 1a (mGluR1a) signaling into the cerebellar cortex [39], a result supported by the already proposed role of metabotropic glutamate receptors (mGluR1a, 2, and 3) in mediating the estradiol effect on functional change (Figure 2) [140,141]. Compelling, mGluR1s is a leading candidate to play a cardinal role in the cerebellar mechanism involved in the regulation of parallel fiber to PCs synapses and in the adaptation of the VOR [26,112]. Besides, it has been shown that the estradiol-mediated modulation of glutamatergic neurotransmission took part at a postsynaptic level and that the GPER signaling was not involved. Further, male mice underwent chronic and systemic administration of the aromatase inhibitor fadrozole showed an impairment of locomotor performance that indirectly indicates a possible role played by nE2 in controlling this cerebellar-influenced motor behavior [39].

Dieni et al., have found that de novo synthesized estradiol acutely regulates cerebellar synaptic plasticity affecting long term potentiation at the parallel fiber-Purkinje cell synapse (PF-LTP) and modulates the encoding of VOR adaptation in adult male rats [40,74]. In these in vitro studies on cerebellar slices, using the aromatase blocker letrozole (LTZ), the authors demonstrated that nE2 specifically affects the PF-LTP acting at postsynaptic level but influences neither the long term depression at the parallel fiber-Purkinje cell synapse (PF-LTD) nor the intrinsic and basal synaptic proprieties of PCs. Further, nE2 seems not to affect the synaptic transmission between climbing fibers and PCs (data not published), a result supported by the previous finding that estradiol does not alter the structure of climbing fibers to PCs synapses [97].

The authors hypothesized that the nE2 effect on cerebellar LTP could be due to either a transient or an acute increase of estradiol synthesis during PF-LTP induction (Figure 4). Indeed, the acute application of the aromatase inhibitor LTZ in cerebellar slices did not influence basal neuronal activity [40] but, the perfusion of estradiol changes the efficacy of glutamatergic transmission at the parallel fiber to PC synapse [39,87].

Furthermore, these effects were probably mainly mediated by the activation of ERβs (due to the much higher ERβs expression than ERαs), as suggested by the fact that estradiol-dependent effects vanished when electrophysiological recordings were performed in the presence of the estrogen receptors antagonist ICI 182,780 (data not published).

Overall, nE2 does not seems essential for basal neurotransmission. Nevertheless, it is reasonable to assume that in the cerebellum estradiol availability may locally and dynamically fluctuate on demand in response to specific signals and that this dynamic nE2 fluctuation may influence in turn basal neurotransmission at parallel fiber-PC synapse if required and affects synaptic plasticity at the same synaptic site [39,40,87]. The fact that in adult rodents was detected not differences in estradiol concentration between plasma and cerebellum tissues in some studies (concentration on average referred in the order of a hundred picomolar) and considerable diversity in estradiol level in cerebellar explants among others may support the assumption of an on-demand fluctuation of nE2 concentration [39,62,84,100,142]. On the other hand, it is well known that phosphorylation processes and glutamatergic, dopaminergic, and calcium signaling can rapidly modify aromatase activity, thereby causing changes in the capability to synthesize nE2 (Figure 3) [3,21,38,143,144,145,146]. Furthermore, activation of motor pathways can locally elevate aromatase activity, and the nE2 concentration may rapidly fluctuate within sensorimotor circuits [21,53,147]. It is worth mentioning that neurotransmitter signaling modulating aromatase activity, and intracellular calcium transient takes part in cerebellar PCs during motor learning phenomena [26,113,115,122]. Thus, potentially, a variety of signals could rapidly and dynamically modulate nE2 synthesis to regulate the estradiol action at the parallel fiber-PC synapse. This scenario implies that classical neurotransmitters and nE2 signaling may influence each other with the consequence that the balance of this cross-influence could be a requisite for the homeostatic control of the synaptic function (Figure 2 and Figure 3).

However, the essential point is that in the in vitro models, the nE2-dependent modulation of parallel fiber-PCs synapses activity is characterized by a low aromatase expression and a localized estradiol synthesis over PCs which modulation is cardinal for the cerebellar dependent learning [39,40,66,84]. Of course, in intact animals, the circulating estradiol, especially when at high plasma level (e.g., during female proestrus), could also contribute to the nE2 effect or interact with it to determine more complex multiple modes and time-course changes in cerebellar network functioning [40,97]. Besides, to date, it has not been possible to rule out the possibility that in physiological conditions, other nearby structures presenting high nE2 synthesis levels, such as inferior olive and hypothalamus, might supply an amount of nE2 to the cerebellum via penetration-diffusion mechanisms [44,83]. The recent discovery of the pineal gland as an active site of the de novo synthesized neurosteroids in birds also adds this endocrine organ closely located to the cerebellum as a potential structure that could contribute to providing estradiol to the cerebellum [148,149].

Consistent with the impact of nE2 on the plasticity of the cerebellar circuitry, it has also been shown that the inhibition of estradiol synthesis by acute and systemic administration of LTZ affects gain increase and decrease in VOR adaptation without influencing basal oculomotor reflexes of adult male rats [40,74] (Figure 4). This finding provides one of the most persuasive evidence indicating a role played by nE2 in regulating adaptive behavior depending on cerebellar plasticity, and diverse observations support it. First, differently than other motor behavior, the VOR depends on a specific cerebellar circuitry and plasticity (Figure 4). Second, cerebellar and vestibular plasticity mediating the VOR adaptation is selectively regulated by nE2 (see below) [40,41,42,92]. Then, in adult male rodents, the blood level of estradiol (often referred to less than a hundred picomolar) is too low to influence, per se, neuronal plasticity and excluding, therefore, a useful contribution of the circulating hormone in regulating cerebellar function for this experimental model [4,44,62,142,150,151].

Moreover, systemically blocking the estradiol synthesis by LTZ produces a rapid impairment in the adaptation of the VOR, which is not consistent with and has a different timescale of the LTZ effect on the depletion of peripherally derived estrogen [74,152]. Further, LTZ, per se, influence neither basal ocular reflexes nor the basal activity of VOR circuitry [40,42,74]. Finally, cerebellar tissue from castrated rodents contains a similar amount of estrogen than intact animals (intact: ~6 pg/mg; castrated: ~9 pg/mg) [39], suggesting an active estradiol synthesis by the cerebellum and possibly nearby regions.

A critical lack for a better understanding of the estradiol action on cerebellar function is the absence of information about a possible effect of nE2 on the glutamatergic neurotransmission within inferior olive, a crucial structure in influencing the activity of cerebellum (Figure 4) and presenting high nE2 synthesis and estradiol receptors (GPR-1 and ERβs) [29,70,89]. The inferior olive computes both GABAergic and glutamatergic signals, and the balance between these two neurotransmitters signalings is an essential factor for the proposed role of the olivocerebellar system in controlling motor coordination [153,154,155,156]. So far, it has been found that endogenous estradiol, as well as an endogenously induced high level of plasma estradiol, exerts a neuroprotective role against a form of cerebellar ataxia experimentally induced through the degeneration of the inferior olivary nucleus in rats [70]. However, to the best of our knowledge, no studies investigated the nE2 influence on synaptic transmission in the inferior olivary nucleus, although exogenous estradiol could affect the pattern discharge in neurons of dorsal accessory olive [157]. Thus, behind the neuroprotective function, the extent to which the estradiol affects the neuronal activity in inferior olive is an unanswered question. Potentially, nE2 might influence cerebellar-dependent motor behaviors also via modulation of neurotransmission within the inferior olivary nucleus.

Estrogens could also contribute to regulating the cerebellar cortex circuitry via modulation of inhibitory GABAergic signaling [158], similar to what happens in other brain regions [53,159]. Due to structural and functional relationships with the cerebellum and the very similar nE2 pathway, vestibular nuclei may be a good model for inferring a potential action of estrogen on GABAergic/glutamatergic neurotransmission balance in the cerebellum. In medial vestibular nuclei slices, estradiol may potentiate the glutamatergic neurotransmission via ERs (ERα and ERβ) activation and induction of intracellular pre and postsynaptic NMDARs-dependent signaling. While a high concentration of exogenous-derived estradiol affects basal neurotransmission, the locally synthesized nE2 plays an essential role only in the LTP induction [42,92]. However, nE2 may regulate the LTP in vestibular nuclei by diminishing the GABAergic neurotransmission as well as enhancing glutamatergic neurotransmission [41]. An action of nE2 on both cerebellar GABAergic and glutamatergic neurotransmission similar to that found in vestibular nuclei is possible and consistent with the nE2-mediated effects discussed in the next section.

### 4.1. Implications for the Impact of nE2 on the Cerebellar Function

The general implication of the findings discussed here is that the cerebellum in adult rodents uses de novo estradiol syntheses to potentiate glutamatergic signaling and regulate cortical synaptic plasticity and related learning phenomena.

In terms of impact on synaptic plasticity, nE2 seems to selectively affect the glutamatergic synapse at parallel fiber-PCs acting on PF-LTP without influencing the PF-LTD. Similarly, nE2 plays a central role in the modulation of the LTP but not the LTD in vestibular nuclei, hippocampus, and striatum [41,54,55,56,92]. The selective effect of nE2 on PF-LTP in the cerebellum is consistent with the unique mechanisms leading to long-term synaptic plasticity at this synaptic site. The free intracellular calcium concentration, along with phosphorylation/dephosphorylation processes within PCs, is the critical factor in determining the polarity of synaptic plasticity [26]. nE2 signaling could facilitate the rapid and moderate increase of intracellular calcium concentration associated with the LTP induction in PCs and consequent to parallel fiber stimulation [22,115,160,161]. Conversely, PF-LTD needs more massive and prolonged cytoplasmatic calcium concentration fluctuation than those requested for the LTP, and it can be produced, per se, through the recruitment of climbing fibers used paired with parallel fiber stimulation for the PF-LTD induction [115,162,163,164]. On the other hand, the calcium-dependent phosphatase/kinase switch determining the polarity of synaptic plasticity may activate the aromatase during PF-LTP by dephosphorylation processes and inactivate it in the case of PF-LTD via phosphorylation (Figure 3) [26,38,76,112,165,166].

Thus, it has been suggested that nE2 fluctuation mediates by aromatase activation/deactivation not only facilitates the LTP but also could prevent that inappropriate calcium increments would switch the LTP in LTD [40].

In the schema proposed here, the brain may use localized nE2 synthesis, independently by the peripheral production of estradiol, to rapidly and selectively regulate cerebellar synaptic plasticity and influence related learning processes that are essential to adapt behavior to changing contexts.

For the behavior mainly considered in this discussion, the VOR (Figure 4), it is mostly accepted that the encoding of gain decrease and increase in the adaptation of this reflex depends on PF-LTP and PF-LTD, respectively [110,112,117,121,122].

However, the observation that nE2 influences both the gain increase and decrease in VOR adaptation but at a cellular level only impacts the PF-LTP suggests that extra-cerebellar, possibly in the vestibular nuclei of the brainstem, as well as cerebellar synaptic plastic sites sensitive to nE2 (Figure 4), should be required for this reflex to adapt [40,41,113,116,117].

Consistent with the “transferred hypothesis” [118,167,168,169], motor memory may be first encoded into the cerebellar cortex and then immediately transferred to vestibular nuclei in a process detectable within minutes after the beginning of the learning process [170]. In this scenario, cerebellar LTP and vestibular LTD would mediate the expression of the VOR adaptive increase, while cerebellar LTD and vestibular LTP might regulate the VOR increase (Figure 4) [40,113,117]. Thus, nE2 may take part in the VOR gain decrease, mainly influencing cerebellar PF-LTP, whereas the gain increase of the reflex would be modulated via nE2-dependent regulation of vestibular LTP (Figure 4) [40,74]. Consistently, nE2 is necessary for LTP induction not only in the cerebellum but even in the vestibular nuclei via induction of potentiation in glutamatergic and inhibition in GABAergic signaling [41,42,91]. Immunohistochemical analysis has already demonstrated the presence of aromatase in the vestibular nuclei of rodents during developing and post-developing periods [91,93]. Furthermore, in adult rats, quantitative real-time PCR measures found similar low aromatase mRNA levels in the brainstem and cerebellum [66]. Similarly, only a few aromatase-immunoreactive neuronal structures were observed in the vestibular system of adult male quail [171]. In the cerebellum and vestibular nuclei, therefore, the maintenance of low aromatase expression during adulthood could have the physiological significance to control, via localized nE2 synthesis, the neurotransmission at a specific synaptic site with particular relevance as those controlling adaptive processes.

Since the aromatase in the cerebellum and brainstem in rodents has very similar expression to that detected in humans, one can also hypothesize similar vestibulo-cerebellar mechanisms controlled by nE2 and leading to motor learning in these two species [66,79,80,83]. A cardinal consequence that extends this concept is that nE2 could influence human perceptive and motor phenomena that require adaptive process and plasticity in the vestibulo-cerebellar networks in physiological as well as pathological conditions [172,173,174,175,176]. Thus, nE2 may exert its influence not only on VOR adaptation but even on the rebalancing after labyrinthine lesion of vestibular reflexes and perception [177,178,179] and on other motor behaviors like balance and locomotion requiring the computational power of cerebellar networks to adapt responses to the context [120,128,180,181]. For instance, the degradation of postural stability observed with age could involve the impairment of the nE2 pathway in the vestibulo-cerebellar circuitry or its interaction with circulating estrogen, as suggested by the improvement of balance in post-menopausal women treated with estrogen [182]. Furthermore, the decrease of locomotory performance in mice after the administration of fadrozole could be a consequence, at least in part, of its effects in blocking the aromatase activity in PCs [39]. Notably, the treatment of breast cancer with aromatase inhibitors has beneficial for the course of the disease; however, side effects might be ataxia, a form of motor coordination impairment typically named dysmetria, and dizziness [183,184]. Nevertheless, it is not possible to rule out that after the administration of aromatase inhibitors, the inhibition of nE2 synthesis in extra-cerebellar structures could contribute to impair the motor function and perception.

Unanswered questions over some aspects related to the cerebellar mechanisms underlying changes in motor performance and behavior could be clarified using the manipulation of estradiol synthesis as a sort of investigation tool. For example, although the synaptic plasticity is known as the primary mechanism on cerebellar dependent motor learning, recent studies on VOR adaptation suggest that also intrinsic neural plasticity may play an essential role in it [117]. However, the relationship between synaptic and intrinsic plasticity in the cerebellum has not been established yet. A possible approach to clarify the latter point could be through the use of estrogens or blockers of their synthesis as it has been shown that estradiol may impact the cerebellar synaptic transmission but not the intrinsic proprieties of neurons in the cerebellum [40,97].

Besides, in the “transferred hypothesis” discussed above, the temporal order of cerebellar and vestibular nuclei synaptic plasticity in the acquisition and maintenance of motor memory is still questioned. Again, to answer this question, one could separately manipulate the estradiol synthesis in the cerebellum and vestibular nuclei in pre, during, and post adaptation. This kind of manipulation, indeed, should specifically affect the LTP at either cerebellar or vestibular nuclei synaptic sites depending on the case [40,41]. If, on the one hand, results from such investigations could help to clarify the temporal relationship between cerebellar and extra cerebellar plastic sites in motor memory formation, on the other hand, they may also be relevant for a better understanding of general mechanisms underlying the cerebellar dependent learning.

### 4.2. The Possible Relationship between Cerebellar nE2 Pathway and Other Brain Structures: Implication for Health

**Figure 5:**
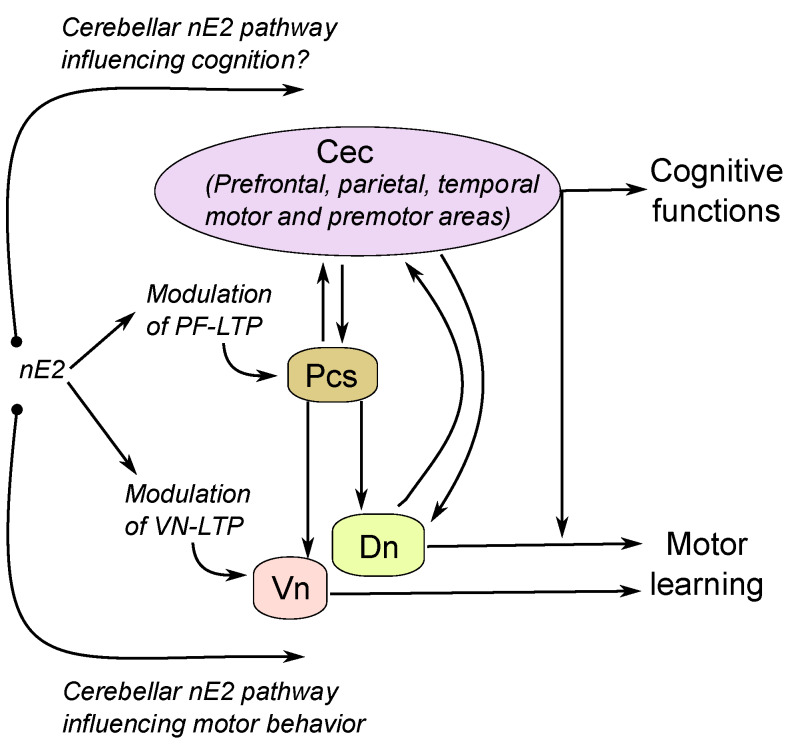
Depiction representing cerebellar nE2 pathways that could influence motor behavior and cognitive functions. Cec, cerebral cortex; Dn, cerebellar deep nuclei; nE2, neurosteroid estradiol; Pcs, Purkinje cells; Pf-LTP, LTP at the parallel fiber-Purkinje cell synapse; Vn, vestibular nuclei; Vn-LTP, LTP in the vestibular nuclei.

Generally speaking, the control of the strength of synapse transmission at parallel fibers to PCs, is essential to regulate the cerebellar cortex output to deep nuclei and extra-cerebellar areas in the brain [120,185,186]. Besides the brainstem, cerebellar communication via closed loops has been found with prefrontal, parietal, temporal, motor, and premotor cortex areas of the cerebral cortex [180,187,188,189]. The cerebellum, therefore, interacts with cortical networks that play a central role in many aspects of cognition, including attention, language, working memory, executive control, temporal representation, emotion, and addiction, as well as the movement [127,128,190]. Moreover, even for the vestibular system has been demonstrated interaction with thalamocortical pathways involved in the cognitive process [191,192,193]. Thus estradiol, including nE2, could widely impact motor and non-motor functions influenced by the cerebellum [120,194] (Figure 5).

There is general agreement that cerebellar output provides to the brain a forward function critical for effective control of movement and cognition [195,196]. The impairment of the forward control should lead to motor dysmetria, a primary cerebellar motor deficit characterized by the loss of the capability to perform rapid and coordinate movements, and for an analogy with the motor domain to cognitive dysmetria [197,198]. The latter indicates an alteration in the synchronous coordination of cognitive capacities leading to inability in controlling thought processes. Not surprisingly, cerebellar dysfunctions, with particular regards to vermis, are linked with schizophrenia and autism spectrum disorder [199,200,201]. Furthermore, schizophrenia and autism spectrum disorder both present dysregulated estrogen pathways during the neurodevelopment of the illness, and adjunctive estradiol therapy has provided a promising outlook in the treatment of schizophrenia [202,203]. Besides, mounting evidence indicates that in Alzheimer’s disease, the cerebellum accumulates Amyloid-β deposits with the consequence of losing PCs and altering cortical synaptic information processing that could contribute to the cognitive and motor deficits that characterize Alzheimer’s disease [132,204]. Concerning the latter issue, it is worthy of mentioning that estradiol may exert neuroprotection against toxic damage induced by Amyloid-β in the cerebellum [205]. Perhaps, despite the intricate estradiol’s interaction with the brain, also the nE2 action in modulating the computational power of the cerebellar cortex could play a significant role in the regulation of the cognitive as well as motor domain. (Figure 5). Further investigations are required for a better understanding of the interaction mechanism between nE2 and the cerebellar function and the possible significance for the cerebellum involvement in sensorimotor and mental processes. Furthermore, additional studies addressing whether sex differences in developing cognitive decline [194,202,203] are influenced by possible diverse interactions between the estrogen hormone and the cerebellar nE2 in males and females are recommended.

## 5. Conclusions

In summary, in contrast with the idea that in the nervous system, the estradiol synthesizing enzyme aromatase requires a high expression to produce useful amounts of nE2, recent investigations in the cerebellum indicate that even a low aromatase expression can be related to the fast nE2 modulation of neuronal physiology. It is now evident that nE2 and not just the gonadal-derived hormone may affect the cerebellar functioning not only during the neonatal life but also in adulthood. The evidence is that in the cerebellum of adult rodents, a brain structure typically characterized by low aromatase expression, nE2 rapidly impacts the glutamatergic neurotransmission at specific synaptic sites as those between the parallel fibers and PCs dendritic spines probably via activation of “non-classical” estradiol signaling in PCs. Furthermore, emerging data indicate that nE2 plays an essential role in the cerebellar-dependent behaviors and motor memory formation, affecting multiple synaptic plastic sites at the cerebellar and possibly extra-cerebellar networks. As an extension of the latter concept, the suggestion is that the nE2 synthesized in the cerebellum (and perhaps in nearby areas) could influence a variety of sensorimotor phenomena depending on cerebellar synaptic plasticity.

Moreover, the fact that the nE2 synthesis influences the computational processes of the cerebellar cortex leads to the consequence that it may even play a role in cognitive as well as motor functions involving the cerebellum. Since altered cortical cerebellar synaptic processing may be involved in different forms of cognitive decline, we believe that the findings reported here may offer absorbing insight into the possibility of developing novel adjunctive estrogen therapy strategies that can be useful in supporting neuroprotection.

## Figures and Tables

**Table 1:** Summary of findings indicating aromatase and estrogen receptors expression and distribution within the cerebellar cortex in rodents.

Cerebellar Cortex Layer	Aromatase		ERα		ERβ		GPER-1	
	Childhood	Adult	Childhood	Adult	Childhood	Adult	Childhood	Adult
Gc	1, 7	1			11, 12, 20	8, 11, 14, 15, 19, 21		18
PC	1, 7	1	11, 20	11	7, 11, 12	11, 8, 14, 15–17, 19, 21		18
Ml					11, 12	11, 19		18
NI	4, 5, 10	2, 3, 4, 9,		3, 6, 13		3, 6		

Data are mainly based on immunohistochemical and mRNA detection techniques. Childhood indicates the period from neonatal life to prepuberty. Adult indicates the period of life starting from peri puberty. ERα, estrogen receptors alpha; ERβ, estrogen receptors beta; Gc, granular cell; GPER-1, G protein estrogen receptors (G protein-coupled transmembrane receptors); Ml, molecular layer; NI, localization not identified (studies only based on mRNA detection techniques); PC, Purkinje cell. 1, Sakamoto et al., 2003 [84]; 2 Tabatadze et al., 2014 [66]; 3 Munetomo et al., 2015 [83]; 4 Lavaque et al., 2005 [81]; 5 Dean et al., 2012 [94]; 6 Abel et al., 2011 [95]; 7 Biamonte et al., 2009 [96]; 8 Andreescu et al., 2008 [97]; 9 Roselli et al. 1984 [82]; 10 Yamada et al. 1994 [98]; 11 Ikeda and Nakai 2006 [88]; 12 Jakab et al. 2001 [99]; 13 Mohamed et al., 2000 [100]; 14 Mitra et al., 2003 [89]; 15 Price and Handa et al. 2000 [90]; 16 Shughrue et al. 1997 [101]; 17 Zhang et al. 2002 [102]; 18 Hazell et al., 2009 [29]; 19 Cardona-Gomez et al. 2000 [103]; 20 Perez et al. 2003 [104]; 21 Shughrue and Merchenthaler 2001 [105]. See the text for detail.

## Abbreviations

nE2	estradiol synthesized in the nervous system
ERs	estrogen receptors
ERα	estrogen receptors isoform α
ERβ	estrogen receptors isoform β
GPCRs	G-protein-coupled receptors
GPER-1	estrogen-G-protein-coupled receptors-1
Gαq-mER	Gαq-membrane estrogen receptor
ER-X	estrogen receptor-X
GABA	Gamma aminobutyric acid
VOR	vestibulo-ocular reflex
PCs	Purkinje cells
BDNF	brain-derived neurotrophic factor; mGluR1a: metabotropic glutamate receptors type 1a
PF-LTP	long term potentiation at the parallel fiber-Purkinje cell synapse
PF-LTD	long term depression at the parallel fiber-Purkinje cell synapse
LTZ	letrozole
ICI 182,780	estrogen receptor antagonist
NMDAR	N-methyl-D-aspartate receptor
LTP	long term potentiation
LTD	long term depression
